# Efficacy of Different Doses and Forms of the GLP-1 Receptor Agonist Semaglutide in Weight Reduction Among Non-diabetic Obese or Overweight Populations

**DOI:** 10.7759/cureus.68786

**Published:** 2024-09-06

**Authors:** Nazeefa Fatima, Abhinav Anand, Aadi R Palvia, Avneet Kaur, Gibran A Azeez, Mounika Thirunagari, Samia Rauf R Butt

**Affiliations:** 1 Clinical Research, California Institute of Behavioral Neurosciences and Psychology, Fairfield, USA; 2 Internal Medicine, California Institute of Behavioral Neurosciences and Psychology, Fairfield, USA; 3 Internal Medicine, Kharghar Medicity Hospital, Navi Mumbai, IND; 4 Pathophysiology, St. George's University, St. George's, GRD; 5 Internal Medicine, Davao Medical School Foundation, Davao City, PHL; 6 General Practice, California Institute of Behavioral Neurosciences and Psychology, Fairfield, USA

**Keywords:** glp-1 receptor agonists, metabolic syndrome (ms), ozempic, semaglutide, treating obesity, weight-loss

## Abstract

Disorders linked to increased body weight are on the rise and obesity is a global epidemic associated with a rising risk for developing comorbidities, such as hypertension or type 2 diabetes. There is a significant need to develop a multimodal approach targeting obesity within clinical medicine. Pharmacological options to produce weight loss have been a popular research area and the novel glucagon-like Peptide-1 receptor agonists (GLP-1 RA) are highly effective glycemic control agents that have shown a substantial weight loss effect. This systematic review explores the efficacy of semaglutide, a GLP-1 RA agent, in a non-diabetic population, looking at endpoints of changes in weight and waist circumference and the percentage of patients achieving a clinically effective weight loss of at least 5%. This study was conducted following the Preferred Reporting Items for Systematic Reviews and Meta-Analyses (PRISMA) 2020 guidelines. A comprehensive search was undertaken to find applicable papers using three databases, including PubMed, PubMed Central, and Cochrane Library. The included articles were narrowed down from an initial pool of 423 papers using filters, automation tools, inclusion/exclusion criteria, and quality appraisal tools. In this systematic review, we have analyzed 10 high-quality studies published in the last five years, including nine randomized control trials (RCTs) and a retrospective cohort study. The aim was to combine the results of these studies, encompassing 6623 participants, to showcase the effectiveness of GLP-1 RAs in the non-diabetic obese or overweight population. The consolidated data from the literature in this systematic review endorses the use of semaglutide as a highly efficient weight-reducing agent, contributing positive insight to both clinicians and researchers in the field of obesity treatment.

## Introduction and background

A significant health and economic burden is linked to the epidemic of increasing body-mass-index (BMI) around the globe. In the United States, seven out of 10 adults are overweight or obese, with a projection of approximately half of the nation's population being obese by 2030 [1]. Those with a BMI greater than or equal to 25 kg/m^2^ are defined as overweight while a BMI of 30 kg/m^2^ or higher is considered obese [2]. Obesity is a chronic and relapsing disease with an extensive list of related comorbidities and increased associated financial liability. The burden of obesity and weight-related complications can add up to an estimated $1429 annually to an individual’s medical expenses in the United States, which is 42% higher than patients of normal weight [3]. In addition to physical disability and psychological load, an increased BMI is linked to numerous metabolic complications, including cardiovascular disease, hypertension, dyslipidemia, obstructive sleep apnea, and non-alcoholic fatty liver disease [3].

Obesity is a significant independent risk factor in the development of type 2 diabetes mellitus (T2DM) as individuals with excess body weight are more prone to insulin resistance and metabolic abnormalities such as hyperinsulinemia, hyperglycemia, and dyslipidemia [4]. Excess adipose tissue results in the release of increased levels of non-esterified fatty acids, glycerol, tumor necrosis factors, leptin, and pro-inflammatory cytokines, which all contribute to insulin resistance [5]. For every kilogram of weight gained, studies have found that the risk of diabetes increases by 4.5% to 9% [5]. This is especially significant for obese individuals as a BMI above 30.0 incurs an estimated 20-times risk of developing T2DM [4]. Therefore, weight loss can improve glucose homeostasis and reduce cardiometabolic risk factors in diabetic patients. In the setting of overweight and obese patients without T2DM, it is even more important to promote weight loss for the prevention of the development of diabetes. As such, weight loss agents are especially clinically significant in non-diabetic overweight and obese patients.

Since the recognition of obesity as a chronic and relapsing disease, obesity management programs have been established by healthcare providers and insurance companies. Historically, lifestyle and behavioral interventions have been at the forefront of promoting weight loss but long-term adherence is the biggest hurdle in its effectiveness in the long-term. Current weight loss guidelines approach weight loss with a multidiscipline approach, combining lifestyle modifications, behavioral therapy, pharmacotherapy, and/or bariatric surgery [2]. Bariatric surgery is the most effective treatment for weight loss and has been shown to decrease mortality from cardiovascular disease by 30% as well as increase overall life expectancy by up to three years [6]. Surgery is, however, not effective in meeting the needs of a global epidemic and carries its own cluster of adverse effects. The hunt for effective and safe weight loss pharmacological agents has been extensive and multiple promising, once-approved anti-obesity medications have been discontinued due to serious adverse effects, leaving only a few behind. In the late 1800s, thyroid extract was one of the first remedies for weight loss, but it resulted in hyperthyroidism, terminating its use as such [6]. Failed anti-obesity agents included sibutramine, fenfluramine, and dexfenfluramine, which all carried cardiovascular side effects [6,7]. Rimonabant, which acted at the cannabinoid receptor, led to increased suicidality while methamphetamine for weight loss had increased addictive potential and drug dependence [7]. Current approved anti-obesity pharmacological options include orlistat, which is a lipase inhibitor; naltrexone/bupropion, an opioid receptor antagonist and noradrenaline reuptake inhibitor; and phentermine/topiramate, an adrenergic agonist/gamma-aminobutyric acid (GABA) receptor modulator [2]. Novel hypoglycemic agents approved for glycemic control in diabetics, glucagon-like peptide-1 receptor agonists (GLP-1 RAs) liraglutide and semaglutide, are also FDA-approved drugs intended for weight management.

Glucagon-like peptide-1 (GLP-1) agents are effective hypoglycemic agents and have the added bonus of weight loss. GLP-1 is an incretin, a naturally occurring hormone secreted by the L-cells of the intestines, that lowers blood glucose through stimulation of insulin secretion and inhibition of gastric emptying [8]. This incretin has also been found to inhibit glucagon secretion, decrease appetite, and increase satiety, resulting in weight loss [8]. Since the approval of liraglutide, Saxenda-3 mg, for weight management of non-diabetic overweight and obese adults in 2014 by the FDA, GLP-1 RAs have created a new field of weight loss agents [5,7]. These GLP-1 agonists include popular brand names Wegovy, Ozempic, Rybelsus to name a few [7]. In contrast to other classes of weight loss agents, which have not exceeded 10% of initial body weight loss at chronic tolerable dose administration, GLP-1 agent semaglutide has shown 14.9% body weight reduction in clinical trials [6]. In June 2021, semaglutide 2.4 mg was approved for chronic weight management by the FDA [7]. It is indicated in adults with obesity or those who are overweight with one comorbidity and is intended to be used in combination with restricting caloric intake and increasing physical activity [7]. Semaglutide comes in oral forms as well as a once-weekly subcutaneous injection in contrast to the once-daily oral agent, liraglutide.

It is important to view the published literature showing the resulting weight loss of semaglutide in obese or overweight adults without diabetes, as the popularity of the drug is at an all-time high with patients and healthcare providers. The FDA currently approves injectable semaglutide for weight loss only under the name, Wegovy, by prescription only for overweight or obese adults as well as children aged 12 or older [6]. With popularity, comes the availability of illegally marketed products and individuals seeking drugs from other markets due to demand. This systematic review will focus on the body weight reduction achieved by GLP-1 RA, semaglutide, in non-diabetic overweight or obese patients.

## Review

Methods

This systematic review follows the guidelines and principles of the Preferred Reporting Items for Systematic Reviews and Meta-Analyses (PRISMA) 2020 [9].

Search Strategy

PubMed, PubMed Central (PMC), and Cochrane Library were used extensively as research databases and search engines to conduct this systematic review. We explored PubMed on March 17, 2024. The research utilized “GLP-1 agonists, Obesity or Overweight, Weight loss.” We used the Boolean terms "OR" and "AND" to combine the relevant concepts with specific keywords, as shown in Table 1

**Table 1:** PubMed search strategy with regular keywords GLP-1 RA: glucagon-like peptide-1 receptor agonists; BMI: body mass index

Concepts	Keywords	PubMed search builder
GLP-1 RA	GLP-1 agonist, Semaglutide	GLP-1 agonist OR Semaglutide
Obesity	Obesity, Overweight, BMI >25, BMI >30	Obesity OR Overweight OR BMI>27 OR BMI>30
Weight loss	Weight loss, Fat loss, Waist Circumference, BMI	Weight Loss OR Total Body Fat Loss OR Waist Circumference OR BMI

Similarly, the same concepts were used as the keywords to create the following Medical Subject Headings Strategy (MeSH). We selected subheadings like therapy, therapeutic use, drug effects, administration and dosage, agonists, etc. The final results are shown in Table 2. 

**Table 2:** MeSH strategy MeSH: Medical Subject Headings Strategy

Keywords	MeSH strategy
Glucagon-Like Peptide-1	("Glucagon-Like Peptides/administration and dosage"[Majr] OR "Glucagon-Like Peptides/agonists"[Majr] OR "Glucagon-Like Peptides/drug effects"[Majr] OR "Glucagon-Like Peptides/therapeutic use"[Majr])
Obesity or Overweight	("Obesity/drug therapy"[Mesh] OR "Obesity/therapy"[Mesh])
Body Weight or Waist Circumference	("Waist Circumference/drug effects"[Mesh] OR "Body Weight/drug effects"[Mesh])

The resulting advanced search strategy, created through the combination of the MeSH terms was, ("Obesity/drug therapy"[Mesh] OR "Obesity/therapy"[Mesh]) AND ("Glucagon-Like Peptides/administration and dosage"[Majr] OR "Glucagon-Like Peptides/agonists"[Majr] OR "Glucagon-Like Peptides/drug effects"[Majr] OR "Glucagon-Like Peptides/therapeutic use"[Majr]) AND ("Waist Circumference/drug effects"[Mesh] OR "Body Weight/drug effects"[Mesh])

Screening of Articles

All the relevant articles were collected, and duplicates were removed. Then, the relevant papers were screened based on the title, abstract, and full-text reading. Finally, 10 research papers were selected and subjected to quality assessment tools.

Inclusion and Exclusion Criteria

The aim of this research is to explore the efficacy of semaglutide, a GLP-1 receptor agonist, in producing weight loss within the non-diabetic overweight and obese populations. This systematic review includes (1) studies involving adults aged 18 years or older, (2) were published as full-text articles, (3) in the English language, and (4) within the past five years. The research articles must include (5) subjects with a BMI in the range of overweight (BMI ≥27 kg/m^2^) or obese (BMI ≥20 kg/m^2^) undergoing and (6) intervention of semaglutide, a GLP-1 RA. Inclusion criteria also consisted of studies that (7) reported endpoints of mean change in body weight (kg/m^2^), change in waist circumference (cm), and total body weight loss (TBWL) percentage. Studies that involved the pediatric and geriatric population were excluded and those that selected only diabetic participants were removed as well. Studies with endpoints that did not include weight loss after intervention were excluded. Literature in languages other than English, and not published within the last five years were excluded.

Results

Study Selection and Quality Assessment

**Figure 1:**
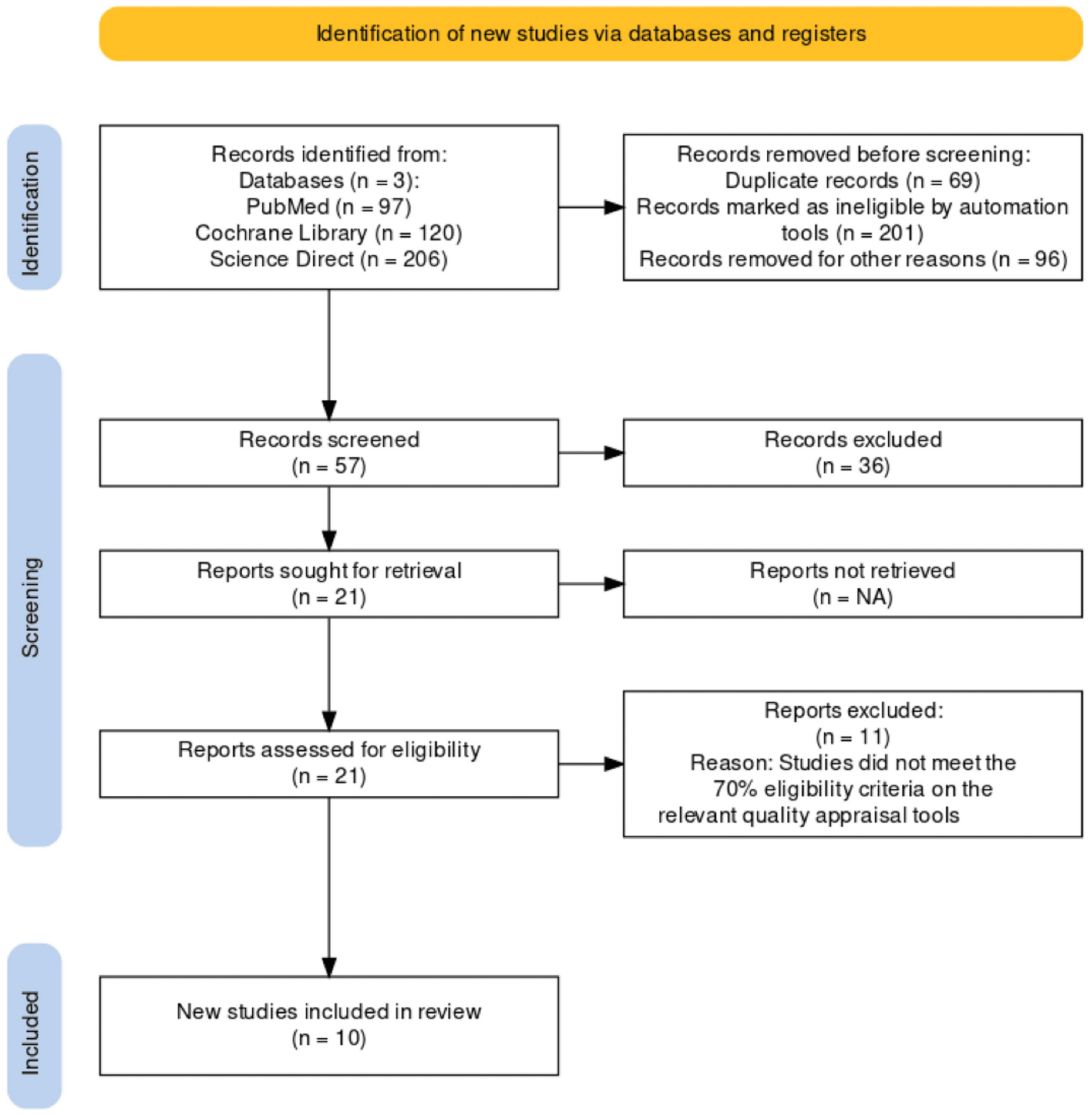
PRISMA flow diagram for included articles PRISMA: Preferred Reporting Items for Systematic Review and Meta-Analyses PRISMA figure adapted from [10].

A multi-step approach was taken according to the PRISMA 2020 guidelines in the study selection process [9]. The initial search of databases resulted in 423 potential articles related to the topic. After applying relevant filters, the search results were narrowed down to 327. Afterward, 69 duplicates and 201 papers were removed by automation tools because of ineligibility, resulting in 57 documents. This number was further reduced to 21 after screening by two independent reviewers based on inclusion/exclusion criteria and relevant title, abstract, and full-text reading. The remaining studies underwent a quality evaluation where they had to achieve at least 70% in quality checks. Eventually, we finalized 10 articles and removed the remaining 11 studies due to poor quality. The complete PRISMA flowchart of our selection algorithm is drawn out in Figure 1.

Table 3 and Table 4 present how each included article was assessed for quality using specific quality appraisal tools. This systematic review includes an observational study as well as randomized control trials (RCTs). For assessing the quality of RCTs, we applied the Cochrane risk of bias (RoB) 2.0 tool, which examines the risk of potential bias in domains including selection, performance, detection, attrition, and reporting of the studies [11]. To assess the quality of observational studies, we used the modified Newcastle-Ottawa Scale (NOS) which evaluates biases in selection, comparability, and outcome [11]. Only those articles were chosen that satisfied >70% of the criteria. The quality of the selected papers using the Cochrane RoB 2.0 tool and the NOS are shown in Tables 3, 4, respectively.

**Table 3:** A quality check of RCTs conducted as per the revised Cochrane bias assessment tool ^+^Low risk ^! ^High risk ^?^Unknown D1: random sequence generation (selection bias); D2: allocation concealment (selection bias); D3: blinding of participants and personnel (performance bias); D4: blinding of outcome assessment (detection bias); D5: incomplete outcome data (attrition bias);  D6: selective reporting (reporting bias) RCTs: randomized controlled trials

Author and year	D1	D2	D3	D4	D5	D6	Overall risk
Garvey et al. (2022) [12]	+	?	+	+	+	+	Low
Kadowaki et al. (2022) [13]	+	+	+	+	+	+	Low
Knop et al. (2023) [14]	+	+	?	+	+	+	Low
Kosiborod et al. (2022) [15]	+	+	+	+	+	!	Low
Mu et al. (2024) [16]	+	+	+	+	+	?	Low
Rubino et al. (2021) [17]	+	+	+	+	+	+	Low
Rubino et al. (2022) [18]	+	+	!	+	+	+	Low
Wadden et al. (2020) [19]	+	+	+	+	+	+	Low
Wilding et al. (2021) [20]	+	+	+	+	+	+	Low

**Table 4:** A quality check of an observational study conducted as per modified NOS ^*^Indicates that the study has met the criteria specified within a particular domain. NOS: Newcastle-Ottawa scale

Modified Newcastle-Ottawa scale		Ghusn et al. 2022 [21]
Selection: (maximum 4 stars)	(1) Representativeness of the exposed cohort	*
	(2) Selection of the non-exposed cohort	
	(3) Ascertainment of exposure	*
	(4) Demonstration that the outcome of interest was not present at the start of the study	*
Comparability: (maximum 2 stars)	(5) Comparability of cohorts on the basis of the design/analysis	*
Outcome: (maximum 3 stars)	(6) Assessment of outcome	*
	(7) Was follow-up long enough for outcomes to occur	*
	(8) Adequacy of follow-up of cohorts	*
Total stars		7/9

Study Characteristics

The included studies included one retrospective cohort study and nine RCTs, conducted from 2015 to 2021, which were published within the last five years of this systematic review. The main outcomes included are average weight change, average change in waist circumference, and percentage of the trial population with a weight loss above 5% to 20%. The characteristics and outcomes of the studies are highlighted in Table 5 and Table 6.

**Table 5:** Study characteristics RCT: randomized controlled trial; STEP: semaglutide treatment effect in people; OASIS: oral semaglutide treatment effect in people with obesity

Author (study ID)	Type	Study name	No.	Baseline	Study duration (weeks)	Study start year
Garvey et al. [12]	RCT	STEP 5	304	Adults with BMI ≥30 or BMI ≥27 with ≥1 weight-related comorbidities, without diabetes	104	2018
Kadowaki et al. [13]	RCT	STEP 6	401	Adults with BMI ≥27 with ≥2 weight-related comorbidities, or BMI ≥35 with ≥1 weight-related comorbidity	68	2019
Knop et al. [14]	RCT	OASIS 1	667	Adults with BMI ≥30 or BMI ≥27 with ≥1 weight-related comorbidities, without diabetes	68	2021
Kosiborod et al. [15]	RCT	-	529	Patients with heart failure with preserved ejection fraction and a BMI ≥30	52	2021
Mu et al. [16]	RCT	STEP 7	375	Adults with BMI ≥30 or BMI ≥27 with ≥1 weight-related comorbidity without or without T2D	44	2020
Rubino et al. [17]	RCT	STEP 4	902	Adults with ≥1 self-reported unsuccessful dietary effort to lose weight and with a BMI ≥30 or BMI ≥27 with ≥1 weight-related comorbidity	68	2018
Rubino et al. [18]	RCT	STEP 8	338	Adults with BMI ≥30 or BMI ≥27 with ≥1 weight-related comorbidities, without diabetes	68	2019
Wadden et al. [19]	RCT	STEP 3	611	Adults with BMI ≥30 or BMI ≥27 with ≥1 weight-related comorbidities, without diabetes	68	2018
Wilding et al. [20]	RCT	STEP 1	1961	Adults with BMI ≥30 or BMI ≥27 with ≥1 weight-related comorbidities, without diabetes	68	2018
Ghusn et al. [21]	Retrospective cohort	-	175	Adults with BMI ≥27 who were prescribed once-weekly semaglutide subcutaneously for ≥3 months	24	2021

**Table 6:** Study results NR: no results

Author	Intervention or matching placebo	Mean weight change (%)	Mean waist circumference (cm)	Percentage of patients with weight loss above:			
				5%	10%	15%	20%
Garvey et al. [12]	Semaglutide 2.4 mg	-14.8	-15.2	77.1	61.8	52.1	36.1
	Placebo	-2.4	-4.3	34.4	13.3	7.0	2.3
Kadowaki et al. [13]	Semaglutide 1.7 mg	-9.6	-7.8	72.4	41.8	24.5	11.2
	Semaglutide 2.4 mg	-13.2	-11.2	82.9	60.6	40.9	19.7
	Placebo	NR	NR	NR			
Knop et al. [14]	Semaglutide 50 mg	-15.1	NR	85	69	54	34
	Placebo	-2.4	NR	26	12	6	3
Kosiborod et al. [15]	Semaglutide 2.4 mg	-13.3	-11.7	NR	65.9	43.9	23.6
	Placebo	-2.6	-2.7	NR	9.5	2.1	0.4
Mu et al. [16]	Semaglutide (initiated at 0.25mg and increased every four weeks until the target dose of 2.4 mg)	-12.1	-10.7	85	63	34	14
	Placebo	-3.6	-3.9	31	10	6	2
Rubino et al. [17]	Semaglutide 2.4 mg (68 weeks)	-7.1	-6.4	88.7	79.0	63.7	39.6
	Semaglutide 2.4 mg (20 weeks) followed by placebo (48 weeks)	+6.1	+3.3	47.6	20.4	9.2	4.8
Rubino et al. [18]	Semaglutide 2.4 mg	-15.8	-13.2	NR	70.9	55.6	38.5
	Liraglutide 3.0 mg	-6.4	-6.6	NR	25.6	12.0	6.0
	Placebo	NR	NR	NR			
Wadden et al. [19]	Semaglutide 2.4 mg	-16.0	-14.6	86.6	75.3	55.8	35.7
	Placebo	-2.4	-4.3	47.6	27	13.2	3.7
Wilding et al. [20]	Semaglutide 2.4 mg	-14.9	-13.54	86.4	69.1	50.5	NR
	Placebo	-2.4	-4.13	31.5	12.0	4.9	NR
Ghusn et al. [21]	Semaglutide 1.7 mg or 2.4 mg	-10.9	NR	87.3	54.9	23.5	7.8

Discussion

Efficacy in weight reduction of variable doses and forms of GLP-1 RA, semaglutide, in a non-diabetic obese or overweight population.

Subcutaneous Semaglutide 2.4 mg

This systematic review includes seven RCTs that assessed treatment with a 2.4 mg subcutaneous dose of semaglutide. Seven of these RCTs were from the Semaglutide Treatment Effect in People with Obesity Program (STEP) and the eighth study included is a 2023 paper by M.N. Kosiborod.

Kosiborod et al. conducted a randomized, double-blind, placebo-controlled trial focusing on the function and symptoms of non-diabetic obese or overweight patients with concurrent heart failure with preserved ejection fraction [15]. This systematic review included this trial since it included patients with a BMI of 30 kg/m^2^ or more, excluded diabetics, and reported on relevant endpoints including TBWL percentage, change in waist circumference as well as the percentage of individuals achieving a weight loss of at least 10%, 15%, and 20% [15]. The intervention was a once-weekly dose of semaglutide 2.4 mg compared to a matching placebo over a period of 52 weeks randomized to a population of 529 individuals [15]. Results revealed an estimated mean body weight loss of -13.3% with the interventional dose and -2.6% with matching placebo [15]. A 62 (23.6%) of 263 patients receiving semaglutide achieved a TBWL of 20% or more while only one (0.4%) of 266 in the placebo group reached the same [15]. This study also reported on the reduction of symptoms of heart failure as well as improvement in physical limitations and exercise function due to the weight loss achieved by patients, suggesting that obesity-related comorbidities such as heart failure would benefit from a GLP-1 RA.

The STEP program intends to establish the use of once-weekly 2.4 mg semaglutide, administered subcutaneously, as a weight loss agent for patients with obesity [20]. The program consists of multiple phase-III, randomized, double-blind, placebo-controlled trials in an overweight or obese population. This systematic review includes STEP trials 1 and 3-8. We excluded the STEP 2 trial as it focused on a population with type two diabetes, where patients were screened for an HbA1c of 7%-10% and/or a diagnosis of T2DM at least six months prior to the study.

STEP 1, 3, 4, 5, and 8 trials all included adults with either a BMI of 30 kg/m^2^ or greater or a BMI of 27 kg/m^2^ or greater with one or more treated or untreated weight-related comorbidities such as hypertension, dyslipidemia, obstructive sleep apnea, or cardiovascular disease, but not diabetes [13-17]. STEP 6 trial included an East Asian adult population from Japan or South Korea, with a BMI of 35 kg/m^2^ or greater with at least one weight-related comorbidity or BMI of 27 kg/m^2^ and greater with at least two weight-related conditions [13]. STEP 6 trial stated that at least one of the comorbidities had to be hypertension, dyslipidemia, or type 2 diabetes, resulting in a population of participants with or without diabetes [13]. The STEP 7 trial recruited a predominantly East Asian population in centers and hospitals from South Korea, China, Hong Kong, and Brazil consisting of a population of diabetics and non-diabetics [16]. Since this trial did not include only a diabetic population, this systematic review has included this trial. Eligibility in this trial included adults diagnosed with diabetes or those without but with a BMI greater than or equal to 30 kg/m^2^ or a BMI of 27 kg/m^2^ or greater with at least one weight-related comorbidity [16].

The intervention in STEP trials 1, 3, and 5 included semaglutide 2.4 mg once weekly or a matching placebo [13-14,16]. In STEP 4, the trial had a crossover design to study the effect of continuous long-term semaglutide vs discontinuation after 20 weeks [17]. Participants were randomized to either receive semaglutide 2.4 mg for the entire 68 weeks or 2.4 mg semaglutide for 20 weeks, followed by placebo for 48 weeks [17]. In the STEP 6 trial, semaglutide was available as a once-weekly subcutaneous injection in a 1.7 mg dose or 2.4 mg dose [13]. The STEP 7 trial initiated the dose of semaglutide subcutaneously once weekly at a dose of 0.25mg, then increased every four weeks to the following doses; 0.5, 1.0, 1.7, until ultimately achieving the maintenance dose of 2.4 mg by week 16 [16]. STEP 8 trial compared the outcome of two different GLP-1 RAs as it involved once-weekly semaglutide 2.4 mg, liraglutide 3.0 mg, or matching placebo [18].

The STEP trials involved additional lifestyle modifications along with the intervention to explore a superior combination of inducing weight loss with the pharmacological agent. Participants in the STEP 1, 4-8 trials received counseling sessions every four weeks to help them adhere to lifestyle interventions [13,15-18,20]. Participants had to follow a reduced calorie diet with a 500-kcal deficit per day and perform 150 minutes of physical activity per week [13,15-18,20]. In the STEP 3 study, partakers received meal replacements in the form of liquid shakes, meal bars, and portion-controlled meals to follow a low-calorie diet of about 1000 to 1200 kcal/day for the first eight weeks of the study [19]. For the remainder of the 68 weeks, participants increased their diet to 1200 to 1800 kcal/day [19]. The trial also mandated that individuals perform 100 minutes of physical activity per week, incrementally increasing to 200 minutes/week [19]. Registered dieticians also provided 30 behavioral counseling sessions across the study duration [19].

Mean body weight change percentages are listed in Table 6. Percent body weight change from baseline to week 68 in the STEP 1 trial was -14.85% compared to -2.41% in the placebo [20]. About 417 (32%) of 1306 patients achieved a weight loss of at least 20% over 68 weeks, compared to -1.7% of 655 in the placebo group [20]. In the STEP 3 trial, the estimated mean weight change from baseline was −16.0% with semaglutide in contrast to -5.7% with placebo [19]. STEP 4 trial reported an estimated mean body weight change at week 68 of −17.4% with continued semaglutide in comparison to −5.0% in the group that received semaglutide for the initial 20 weeks, followed by placebo for the remaining 48 weeks [17]. During the first 20 weeks, all groups receiving semaglutide had a decline in mean body weight by -10.6% [17]. Following the crossover, the group that continued semaglutide had an estimated -7.9% weight change from weeks 20 to 68 while the group that stopped taking the drug and were switched to placebo for the remainder of the study reported a mean body weight change of +6.9% [17]. STEP 5 is the longest-duration trial included in this systematic review, lasting 104 weeks compared to the other STEP trials, which allowed the STEP 5 study to report outcomes over a longer, two-year period [12]. The mean change in body weight at week 104 was −15.2% in the intervention group versus −2.6% with placebo [12]. In the STEP 6 trial, the mean reduction in body weight observed was -9.6% on 1.7 mg, -13.2% on 2.4 mg, and -2.1% with placebo [13]. The mean weight reduction observed at the conclusion of the STEP 7 trial was -12.1% in the intervention group and -3.6% with placebo. Finally, the STEP 8 trial reported a mean weight change of -15.8% with semaglutide, -6.4% with liraglutide, and -1.9% with placebo [18].

Semaglutide 1.7 mg or 2.4 mg in Clinical Practice

This systematic review includes one observational study carried out using electronic medical record data from patients undergoing Semaglutide treatment in a clinical setting. Ghusn et al. conducted a retrospective cohort study using data collected at a referral center for weight management [21]. 175 patients were included based on the criteria of BMI of 27 kg/m^2^ or more, receiving once-weekly semaglutide 1.7 mg or 2.4 mg subcutaneously for three months or more [21]. Researchers excluded those with a history of bariatric surgery or who were taking other anti-obesity medications simultaneously [21]. The TBWL percentage achieved was -5.9% at three months and -10.9% at six months [21]. These results showed that weight loss achieved through clinical doses of semaglutide in the real-life setting was similar to those of RCTs. The study also reported that the mean weight loss achieved was higher in patients without diabetes, reporting -6.3% TBWL at 12 weeks (vs. -3.9% in diabetics) and -11.8% at 24 weeks (vs. -7.2% at six months) [21]. Follow-up at 24 weeks confirmed that eight (7.8%) patients out of 102, achieved weight loss of at least 20% [21]. These results highlight the efficacy of the GLP-1 agent as a treatment of obesity and solely as a weight loss agent. It'll be imperative to conduct future cohort studies with longer follow-up periods to see results comparable to clinical trials. Furthermore, clinical trials are conducted in conjunction with controlled lifestyle interventions which electronic medical records cannot confirm.

Oral Semaglutide 50 mg

Knop et al. conducted the oral semaglutide treatment effect in people with obesity (OASIS) 1 trial, a randomized, double-blind, placebo-controlled trial with the intervention of oral dose of semaglutide that was incrementally increased to 50 mg [14]. 667 participants were randomized based on the criteria of being non-diabetic and having a BMI of 30 kg/m^2^ or greater, or at least 27 kg/m^2^ with a body weight-related complication [14]. These complications could be treated or untreated and included hypertension, dyslipidemia, obstructive sleep apnea, or cardiovascular disease. This trial demonstrated the greatest magnitude of weight loss achieved by an oral anti-obesity agent to date. Over the course of 68 weeks, once-daily oral 50 mg semaglutide resulted in an estimated mean body weight change of 15.1%, compared to -2.4% via matching placebo [14]. This trial was the only RCT in this systematic review that used an oral form of semaglutide as the intervention. 107 (34%) of 317 participants achieved a weight loss of at least 20%, compared to eight (3%) out of 295 from the placebo group [14]. The OASIS is a clinical development program consisting of four trials intended for obese or overweight individuals with an oral 25 mg or 50 mg semaglutide [14]. Further studies comparing the oral forms to the injectable would help establish which variation is better for certain patient populations and also compare the adverse effect profile of the two forms.

## Conclusions

In conclusion, this systematic review synthesizes the published literature on the efficacy of varying doses and forms of the GLP-1 RA, semaglutide. Findings from the included literature indicate the effectiveness of semaglutide in producing weight reduction across studies when compared to placebo. Clinically effective weight loss, defined as 5% weight loss, was achieved by a percentage of trial populations ranging from 82.9% to 88.7% on once-weekly semaglutide 2.4 mg. Dose-dependent reduction in body weight was observed in study durations ranging from 52 to 104 weeks and explored both, oral or injectable formulations. This review enforces the use of the GLP-1 RA pharmacotherapy for a resulting weight loss benefit in overweight or obese patients without concurrent diabetes. Further studies following cohorts of real patients undergoing treatment will help clinicians design favorable regimens for optimal efficacy and adherence in the future. Additionally, longer trials or observational studies are required to demonstrate the extended benefits versus adverse effects profile of these agents.

## References

[1] Ward ZJ, Bleich SN, Long MW, Gortmaker SL (2021). Association of body mass index with health care expenditures in the United States by age and sex. PLoS One.

[2] Idrees Z, Cancarevic I, Huang L (2022). FDA-approved pharmacotherapy for weight loss over the last decade. Cureus.

[3] Apovian CM (2016). Obesity: definition, comorbidities, causes, and burden. Am J Manag Care.

[4] Guo X, Zhou Z, Lyu X (2022). The antiobesity effect and safety of GLP-1 receptor agonist in overweight/obese patients without diabetes: a systematic review and meta-analysis. Horm Metab Res.

[5] Liao C, Liang X, Zhang X, Li Y (2023). The effects of GLP-1 receptor agonists on visceral fat and liver ectopic fat in an adult population with or without diabetes and nonalcoholic fatty liver disease: A systematic review and meta-analysis. PLoS One.

[6] Müller TD, Blüher M, Tschöp MH, DiMarchi RD (2022). Anti-obesity drug discovery: advances and challenges. Nat Rev Drug Discov.

[7] Kloock S, Ziegler CG, Dischinger U (2023). Obesity and its comorbidities, current treatment options and future perspectives: challenging bariatric surgery?. Pharmacol Ther.

[8] Aldawsari M, Almadani FA, Almuhammadi N, Algabsani S, Alamro Y, Aldhwayan M (2023). The efficacy of GLP-1 analogues on appetite parameters, gastric emptying, food preference and taste among adults with obesity: systematic review of randomized controlled trials. Diabetes Metab Syndr Obes.

[9] Page MJ, Moher D, Bossuyt PM (2021). PRISMA 2020 explanation and elaboration: updated guidance and exemplars for reporting systematic reviews. Br Med J.

[10] Haddaway NR, Page MJ, Pritchard CC, McGuinness LA (2022). PRISMA2020: an R package and Shiny app for producing PRISMA 2020-compliant flow diagrams, with interactivity for optimised digital transparency and Open Synthesis. Campbell Syst Rev.

[11] Farrah K, Young K, Tunis MC, Zhao L (2019). Risk of bias tools in systematic reviews of health interventions: an analysis of PROSPERO-registered protocols. Syst Rev.

[12] Garvey WT, Batterham RL, Bhatta M (2022). Two-year effects of semaglutide in adults with overweight or obesity: the STEP 5 trial. Nat Med.

[13] Kadowaki T, Isendahl J, Khalid U (2022). Semaglutide once a week in adults with overweight or obesity, with or without type 2 diabetes in an east Asian population (STEP 6): a randomised, double-blind, double-dummy, placebo-controlled, phase 3a trial. Lancet Diabetes Endocrinol.

[14] Knop FK, Aroda VR, do Vale RD (2023). Oral semaglutide 50 mg taken once per day in adults with overweight or obesity (OASIS 1): a randomised, double-blind, placebo-controlled, phase 3 trial. Lancet.

[15] Kosiborod MN, Abildstrøm SZ, Borlaug BA (2023). Semaglutide in patients with heart failure with preserved ejection fraction and obesity. N Engl J Med.

[16] Mu Y, Bao X, Eliaschewitz FG (2024). Efficacy and safety of once weekly semaglutide 2·4 mg for weight management in a predominantly east Asian population with overweight or obesity (STEP 7): a double-blind, multicentre, randomised controlled trial. Lancet Diabetes Endocrinol.

[17] Rubino D, Abrahamsson N, Davies M (2021). Effect of continued weekly subcutaneous semaglutide vs placebo on weight loss maintenance in adults with overweight or obesity: the step 4 randomized clinical trial. J Am Med Assoc.

[18] Rubino DM, Greenway FL, Khalid U (2022). Effect of weekly subcutaneous semaglutide vs Daily liraglutide on body weight in adults with overweight or obesity without diabetes: the step 8 randomized clinical trial. J Am Med Assoc.

[19] Wadden TA, Bailey TS, Billings LK (2021). Effect of subcutaneous semaglutide vs placebo as an adjunct to intensive behavioral therapy on body weight in adults with overweight or obesity: the step 3 randomized clinical trial. J Am Med Assoc.

[20] Wilding JP, Batterham RL, Calanna S (2021). Once-weekly semaglutide in adults with overweight or obesity. N Engl J Med.

[21] Ghusn W, De la Rosa A, Sacoto D (2022). Weight loss outcomes associated with semaglutide treatment for patients with overweight or obesity. JAMA Netw Open.

